# A Short-Term Intervention of High-Intensity Exercise and Anodal-tDCS on Motor Learning in Middle-Aged Adults: An RCT

**DOI:** 10.3389/fnhum.2021.661079

**Published:** 2021-06-16

**Authors:** Clare Quinlan, Ben Rattray, Disa Pryor, Joseph M. Northey, James Coxon, Nicolas Cherbuin, Sophie C. Andrews

**Affiliations:** ^1^UC Research Institute for Sport and Exercise, University of Canberra, Canberra, ACT, Australia; ^2^Discipline of Sport and Exercise Science, Faculty of Health, University of Canberra, Canberra, ACT, Australia; ^3^Centre for Research on Ageing, Health and Wellbeing, Australian National University, Canberra, ACT, Australia; ^4^Turner Institute for Brain and Mental Health, School of Psychological Sciences, Monash University, Clayton, VIC, Australia; ^5^Neuroscience Research Australia, Randwick, NSW, Australia; ^6^School of Psychology, University of New South Wales, Sydney, NSW, Australia

**Keywords:** cognition, non-invasive brain stimulation, motor cortex, aerobic exercise, transcranial direct current stimulation

## Abstract

High-intensity exercise has enhanced motor learning in healthy young adults. Anodal-transcranial direct current stimulation (a-tDCS) may optimize these effects. This study aimed to determine the effects of a short-term high-intensity interval exercise intervention either with or without a-tDCS on the learning and retention of a novel motor task in middle-aged adults. Forty-two healthy middle-aged adults (age = 44.6 ± 6.3, female = 76%) were randomized into three groups: exercise and active a-tDCS, exercise and sham a-tDCS, and a non-exercise and sham a-tDCS control. Participants completed a baseline testing session, followed by three intervention sessions 48-h apart. The exercise groups completed 20-min of high-intensity exercise followed by a novel sequential visual isometric pinch task (SVIPT) while receiving 20-min of 1.5 mA a-tDCS, or sham tDCS. The control group completed 20-min of reading before receiving sham a-tDCS during the SVIPT. Learning was assessed by skill change within and between intervention sessions. Participants returned 5–7 days after the final intervention session and performed the SVIPT task to assess retention. All three groups showed evidence of learning on the SVIPT task. Neither group displayed enhanced overall learning or retention when compared to the control group. High-intensity exercise with or without a-tDCS did not improve learning or retention of a novel motor task in middle-aged adults. The methodological framework provides direction for future research to investigate the potential of differing exercise intensity effects on learning and retention.

## Introduction

Aging is associated with declines in motor function and control which can cause increased cognitive effort in functional tasks (Seidler et al., 2010), as well as difficulty maintaining employability and safety in the aging workforce (Kowalski-Trakofler et al., 2005). Thus, the development of strategies which optimize the acquisition and retention of motor-skills are crucial in maintaining motor function and enabling healthy aging. Middle adulthood is a critical window for interventions targeted at delaying the onset of symptom-related cognitive decline (Macpherson et al., 2017), and may be an appropriate time to target motor-skill learning.

Participating in exercise can have positive benefits on brain function, dependent on the type of exercise, as well as the cognitive domain targeted (Mandolesi et al., 2018). Compared to low intensity exercise, which refers to exercise at 28–39% of VO_2max_, or 45–54% of a person’s maximum heart rate (%HRmax), high intensity exercise can range from 60% of VO_2max_ and 70% HRmax to 100%, representing a maximal effort (Vanhees et al., 2012; Buchheit and Laursen, 2013). High-intensity interval exercise consists of repeated efforts of exercise ranging from less than 45 s to 2–4 min efforts (Buchheit and Laursen, 2013), For motor learning, a single session of high-intensity-interval exercise can benefit learning between sessions, when performed 20 min after learning, more than lower intensity exercise (Thomas et al., 2016). Exercise performed approximately 20 min before learning has also been observed to increase motor skill learning (Mang et al., 2014), and consolidation (Stavrinos and Coxon, 2017). High-intensity exercise performed immediately prior to task practice (Skriver et al., 2014), and following task practice (Roig et al., 2012) resulted in higher retention at 7 days compared to non-exercise control groups. However, this has not yet been investigated in middle-aged adults (35–55 years). High-intensity exercise may contribute to a cascade of neuromodulators beneficial to increased neuronal plasticity and long-term potentiation (Loprinzi et al., 2019). This may include a transient increase in brain-derived neurotrophic factor (BDNF) which appears to display a dose-response relationship with exercise intensity, in which high-intensity exercise increases BDNF above that of continuous exercise (Saucedo Marquez et al., 2015). Further, high-intensity exercise can increase lactate, which crosses the blood-brain barrier, and can enhance synaptic plasticity (Yang et al., 2014). High-intensity exercise has also been shown to increase the neuroplastic response to intermittent theta burst stimulation more so than moderate-intensity exercise in healthy adults (Andrews et al., 2020). Supporting this, a recent meta-analysis of 22 studies investigating the effects of acute cardiovascular exercise on motor learning and memory task performance (Wanner et al., 2020), concluded that high intensity exercise could be beneficial to motor memory consolidation.

Optimization of the benefits of exercise may occur by its combination with other potential neuro-enhancing methods, such as non-invasive brain stimulation. It is posited that transcranial direct current stimulation (tDCS) can be used to alter neuronal excitability. Anodal-tDCS (a-tDCS) is thought to increase cortical stimulation by subthreshold depolarization of neurons in the target region of stimulation. This has been demonstrated in pharmacological investigations showing elimination of excitability effects of a-tDCS when voltage-gated sodium channels are blocked, and a reduction in excitability when calcium channels are blocked (Nitsche et al., 2003a). Although the precise mechanisms are not fully understood (Bestmann et al., 2015), a-tDCS may enhance cognitive function by increasing the chance of neuronal firing. However, there is increasing recognition that these responses may be highly variable due to dose administration, and biological responses to the administered stimuli (Li et al., 2015). On its own, when applied during a motor task, a number of studies have demonstrated that a-tDCS has enhanced the learning and consolidation of a motor task (Nitsche et al., 2003b; Reis et al., 2009), as well as the rate of learning compared to c-tDCS (Stagg et al., 2011). Additionally, a-tDCS may act to optimize the effects of exercise on cognitive training outcomes through shared mechanisms (Ward et al., 2017; Steinberg et al., 2019), and this synergistic benefit may also be seen in the context of motor learning. One such mechanism, may be the shared involvement of BDNF. BDNF has been implicated in long-term potentiation and long-term memory formation (Cunha et al., 2010), exemplified by “rescue protocols,” in which treatment with BDNF largely revered the synaptic deficits observed in BDNF knockout mice brain (Pozzo-Miller et al., 1999). As described above, exercise increases BDNF in a dose response manner (Schmolesky et al., 2013; Saucedo Marquez et al., 2015) with high-intensity exercise increasing BDNF more so than moderate intensity exercise (Schmolesky et al., 2013). As BDNF has been shown to be a key modulator in long-term potentiation induced by direct current stimulation (Fritsch et al., 2010), it has been posited these pathways may converge allowing for the increase in BDNF following exercise to form an optimal environment for successful tDCS induced long-term potentiation (Steinberg et al., 2019). In this instance, exercise may act as a primer, rendering the synapses more responsive to future stimulation (Kronberg et al., 2017; Steinberg et al., 2019).

In young adults, combining both a high-intensity cardiovascular and resistance exercise protocol and a-tDCS within the same intervention, resulted in greater improvements on tests of intelligence compared to either intervention alone in a 4-month randomized controlled trial, however, the timing between intervention components was not described (Ward et al., 2017). High-intensity interval exercise may be more effective in enhancing neuroplasticity induced by brain stimulation when performed prior to, instead of after, a brain stimulation protocol (Mellow et al., 2020). To date, the investigators are not aware of any published research investigating the combined effects of exercise and a-tDCS on motor-skill learning. Positive effects of a single session of high-intensity exercise on motor-skill learning and retention (Roig et al., 2012; Mang et al., 2014; Thomas et al., 2016; Stavrinos and Coxon, 2017) have been demonstrated in healthy young adults. However, to date, the effects of high-intensity interval exercise, over multiple days has not been investigated. While it has been hypothesized that the effects of a-tDCS and exercise may be additive to improving cognitive enhancement (Steinberg et al., 2019), this has not been investigated in motor-learning. This study aimed to investigate the hypothesis that a short-term (3 day) high-intensity interval exercise intervention would enhance motor-skill learning and retention over multiple days compared to no exercise in middle-aged adults, and that the benefits would be further enhanced through combination with a-tDCS applied during the completion of the motor learning task.

## Materials and Methods

### Participants

Participants aged 35–55 were recruited through the distribution of flyers, social media, and word of mouth. Included participants had no known history of neurological impairment, no current use of psychoactive medications (e.g., anti-depressants), no contraindications to tDCS (DaSilva et al., 2011; Antal et al., 2017), no diagnosed color blindness, had 20/20 corrected vision, and were safe to undertake high-intensity exercise, as assessed by a pre-exercise screening questionnaire [Adult Pre-Screening Exercise Tool VI (2011), ESSA]. The Goldberg Anxiety and Depression Scale (Goldberg et al., 1988) was used to assess participants’ self-reported depression and anxiety symptoms to ensure homogeneity across all groups at baseline. Forty-two participants (age = 44.6 ± 6.3, female = 32, left handed = 3), were included in the final analysis. Participants were considered physical active, on average reporting 512 ± 675 min of moderate-vigorous physical activity (MVPA) per week.

**FIGURE 1:**
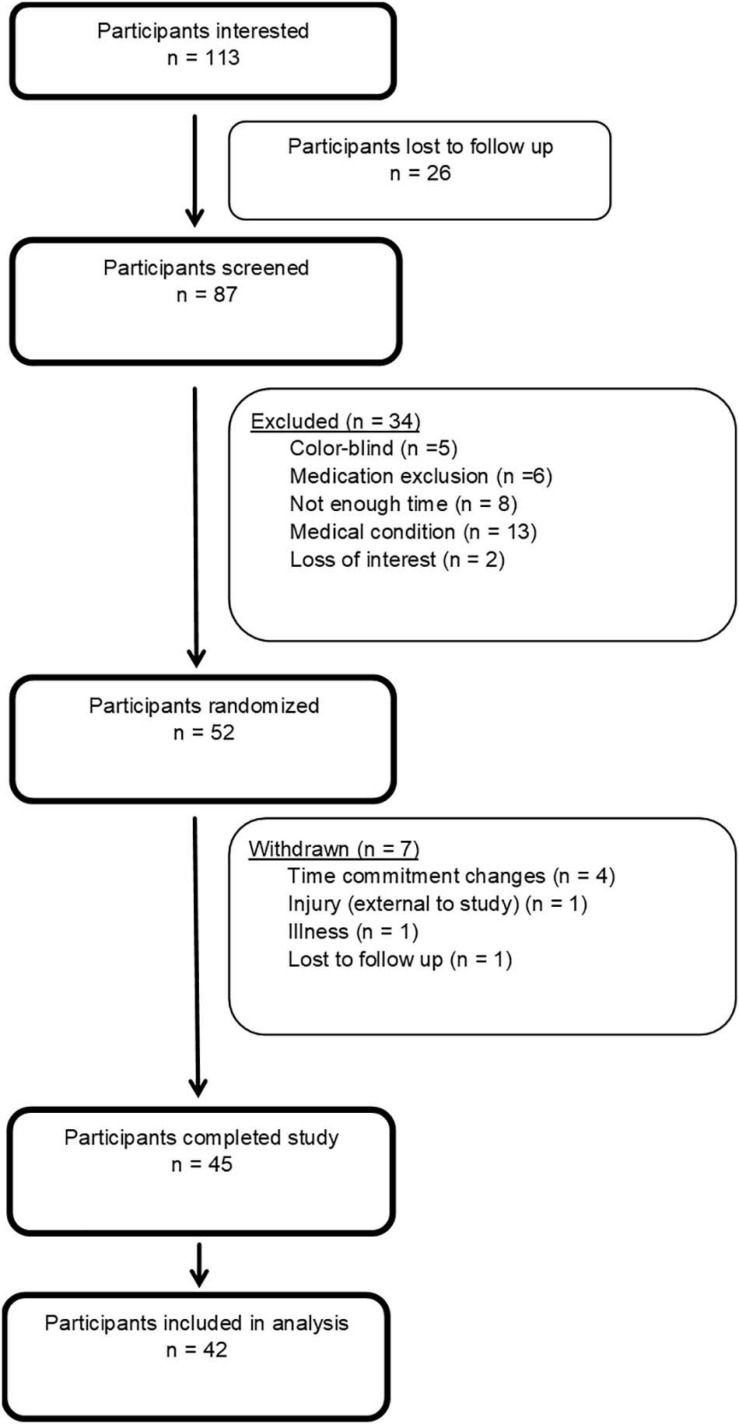
Flow of participants through study.

In consultation with a statistician, and using validated online power-analysis software (Schoenfeld, 2015), an a priori sample size of 42 participants was estimated would provide sufficient power to detect significant differences (α = 0.05; β = 0.80) for a three-condition study with repeated measures. This power analysis was calculated using findings from a previous study that has shown an estimated mean difference in skill of 0.19 ± 0.11 on the same motor task, with a similar exercise protocol as this study, in young adults (Stavrinos and Coxon, 2017). Based on previous experience, we aimed to recruit a minimum of 51 participants to allow for a 10% drop out rate, and a 10% exclusion rate for equipment errors resulting in missing data. Forty-five participants completed the full study protocol, however, three were excluded due to concerns with recording error (*n* = 2) and tDCS application (repeated impedance errors during a sessions) (*n* = 1), As such, 42 participants were included in the analysis (see Figure 1).

The study protocol was approved by the University of Canberra and the Australian National University Human Research Ethics Committee (HREC 16-121, and 2016/322, respectively) and all participants provided written informed consent before commencement.

### Study Design

**FIGURE 2:**
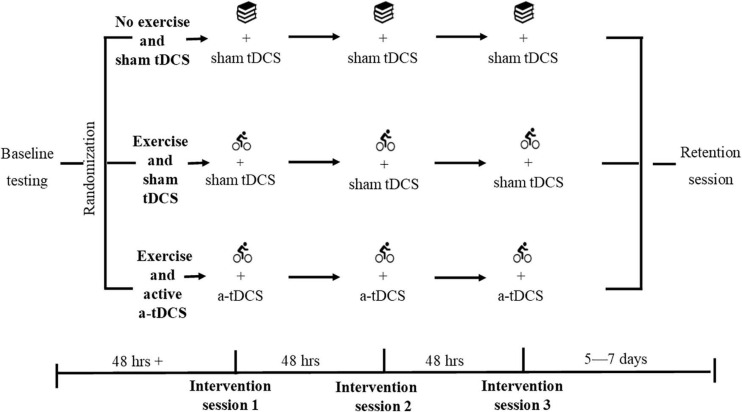
Study design. Participants were randomized following standardized baseline testing. Each group then completed their assigned protocol at each of the three intervention sessions. The exercise and active a-tDCS group performed 20 min of cycling, followed by the learning task with active a-tDCS. The exercise and sham tDCS group performed 20 min of cycling, followed by the learning task with sham tDCS. The non-exercise and sham tDCS group performed 20 min of quiet reading, followed by the learning task with sham tDCS. The learning task and relevant tDCS protocol were applied within 10 min of the completion of exercise. All participants then completed a standardized retention session 5–7 days later.

A three-arm randomized-controlled trial was conducted. On completion of screening and written informed consent, participants were randomized to one of three experimental groups stratified for sex: (1) an exercise group with sham (inactive) tDCS, (2) an exercise group with active a-tDCS, or (3) a no exercise (control) group with sham tDCS. The randomization was completed by a researcher external to the data collection process, utilizing an online random number generator (randomizer.org). Double blinding of the tDCS protocol was applied to all participants, and the researchers involved in data collection until after data were cleaned for analysis. All participants attended the laboratory to complete a standardized baseline session before returning at least 48-h later to complete three intervention sessions over 5 days, with 48-h between sessions (Figure 2). Participants completed a standardized retention session 5–7 days later. Participants were asked to refrain from strenuous exercise, caffeine, and alcohol in the 24-h before all testing sessions.

### Procedures

#### Baseline Testing

During baseline testing, participants’ height, weight, anxiety and depression symptomatology were recorded. Participants then completed a task of manual dexterity, a short battery of cognitive tasks, and a graded exercise test on a cycle ergometer (High-Performance Ergometer, Schoberer Rad MeBtechnik, Germany).

#### Intervention Sessions

##### Exercise and Active a-tDCS

Participants exercised for 20-min on a cycle ergometer (High-Performance Ergometer, Schoberer Rad MeBtechnik, Germany). The protocol shared similarities to previous studies (Roig et al., 2012; Mang et al., 2014; Stavrinos and Coxon, 2017). Participants completed a 4-min warm-up at 50 W, except in two instances where, for participant comfort, due to exceptional fitness 80 W was used, and one instance of low fitness 20 W was used, at a self-selected cadence. Participants then completed four 2-min epochs of cycling at 90% of the peak load achieved during the graded exercise test at a cadence of 90 revolutions per minute (RPM) interspersed by 2-min of active recovery at 50 to 80 W, to accommodate participant comfort, and self-selected cadence. Heart rate (Polar Electro, Kempele, Finland) and rating of perceived exertion (6–20 grade scale, Borg) were monitored before and after each high-intensity epoch.

Within 10 min following the exercise, participants were fitted with a STARSTIM (Neuroelectrics, Barcelona, Spain) tDCS cap with two saline-soaked 25 cm^2^ sponge-based electrodes. Participants received 20-min of 1.5 mA bi-hemispheric a-tDCS with a 30-s ramp-up and ramp-down period. The anodal stimulation applied over the dominant motor cortex (M1) (C3/C4), determined by participant handedness, which has been previously shown to increase acquisition of motor movements, and the cathodal return electrode situated over the non-dominant M1 (Waters-Metenier et al., 2014). Treatments were administered in a double-blind manner. During this 20-min, participants completed four blocks of a motor-skill task: the SVIPT.

##### Exercise and Sham tDCS

Participants in this group undertook the same exercise protocol as above. The only difference was following the exercise participants received sham-tDCS. The electrical current was only administered for the first and last 30 s.

##### Non-exercise and Sham tDCS

Participants in this group completed the same sham tDCS protocol as the Exercise and sham tDCS group above, however, 20-min of quiet reading replaced the exercise.

#### Retention Assessment

5–7 days after the final intervention sessions, all participants returned to complete two blocks of the SVIPT task to measure skill retention. The task was preceded with 20-min of quiet reading to ensure all participants were at a similar level of arousal prior to testing. No exercise, a-tDCS, or sham tDCS was administered during this session. Participants reported which tDCS protocol they thought they received during their intervention sessions.

## Measures

### Primary Outcome Measure

**FIGURE 3:**
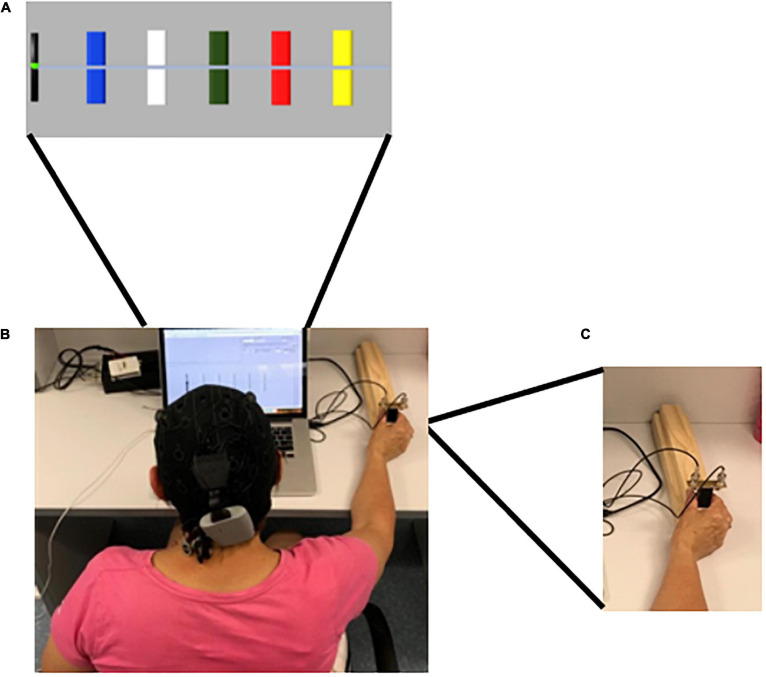
Sequential visual isometric pinch task (SVIPT). **(A)** Representation of SVIPT task presented on screen to participants. The Colors were presented from left to right in the order BLUE – WHITE – GREEN – RED – YELLOW. Participants were instructed to hit the targets in the order RED – BLUE – GREEN – YELLOW – WHITE, returning to the (black) home tab between each. **(B)** tDCS cap with electrodes. Participants randomized into an active-tDCS group were fitted with a tDCS cap with two saline-soaked 25 cm^2^ sponge-based electrodes. 20-min of 1.5 mA bi-hemispheric a-tDCS with the anodal stimulation applied over the dominant motor cortex (M1) (C3/C4), determined by participant handedness. For participants randomized to the sham-tDCS group, a 30 s ramp period was applied at the beginning and the end of the 20-min period only. **(C)** The cursor was moved between the home tab to each of the required colors in a horizontal motion by pinching the force transducer between the thumb and the first finger of the dominant hand.

The primary outcome measure was motor-skill learning assessed by the performance on a SVIPT (Reis et al., 2009), which was novel to all participants. The implementation of the SVIPT was similar to previous reports (Reis et al., 2009; Stavrinos and Coxon, 2017), utilizing MATLAB software (MATLAB 2016a, The MathWorks, Inc., MA, United States). Participants received standardized instructions and familiarization to the SVIPT presented both visually and verbally. Participants were seated at a laptop computer, with a 15-inch screen, and held a force transducer with the thumb and first finger of their dominant hand, determined by self-report, keeping their digits straight. Pinching the force transducer created force pulses which moved the cursor on the screen, from a black (home) tab at the left of the screen, in a horizontal motion to the right. The targets changed from blank to colored to indicate the beginning of the trial, appearing on screen from left to right in the order BLUE – WHITE – GREEN – RED – YELLOW. Participants were instructed to navigate the cursor from the home tab to the five colored targets, displayed in Figure 3, returning to the home tab in between each target in the order RED – BLUE – GREEN – YELLOW – WHITE, emphasizing speed and accuracy equally, with each repetition a single trial. A visual reminder of the order was presented above the laptop during all trials. This order was consistent across all trials and participants (Reis et al., 2009). There were eight trials per block, and four blocks per intervention session and two blocks in the retention session. Visual feedback was provided after each block through a graphic representation of the average speed and accuracy of the completed block, and written and verbal feedback comparing the current block to the previous block, which could not be skipped by participants. The target centers were set to 8.75, 17.5, 26.25, 35, and 43.75 percent of the participants maximal voluntary contraction (MVC), respectively. MVC was determined prior to the familiarization trial, which took place on intervention day one, prior to the intervention session, by participants briefly pinching the force transducer, as described above, at their maximal capacity. The highest MVC of three attempts was used. For familiarization, participants completed two blocks of the learning task. Motor-skill was determined by calculating a skill measure, as described previously (Reis et al., 2009; Stavrinos and Coxon, 2017). Task performance of the SVIPT consisted of three components (1) online motor-skill change: assessed by comparing the average motor-skill of each block throughout the learning phase, (2) offline motor-skill change: assessed by assessing the average motor-skill level of the final block of each intervention session to that of the first block of the proceeding session, and (3) retention of motor-skill: assessed by comparing the average motor-skill from the final intervention session, to that of the two retention blocks.

### Participant Descriptives

#### Depression and Anxiety Symptomatology

Symptoms of depression and anxiety may cause disturbances in motor-skill learning (Caligiuri and Ellwanger, 2000). Depression and anxiety symptomatology were assessed using the Goldberg Anxiety and Depression Scale (Goldberg et al., 1988) to allow for group comparison.

#### Manual Dexterity

To allow for the comparison of participants manual dexterity at baselines, participants completed the first three stages of the Purdue Pegboard task (Lafayette Instrument Company, 2015). Participants inserted as many pegs into the board within a 30-s period, with their dominant hand, their non-dominant hand, and finally with both hands together.

#### Cognitive Function

A battery of computerized cognitive tasks was used to test group similarity at baseline. The battery assessed working memory, attention, processing speed, set-shifting, and inhibitory control as these domains may play an increased role in motor functioning with age (Ren et al., 2013). All tasks were designed and administered using E-Prime 3.0 software (Psychology Software Tools, Pittsburgh, PA, United States).

Working memory was assessed using a Sternberg task, consisting of trials with a stimulus encoding phase and memory retrieval phase (Kober et al., 2016). Selective attention was assessed using a Stroop task (Stroop, 1935), with a working set of 102 trials, with 70% incongruent trials. A Go/No-Go task employing two task levels of increasing difficulty assessed attention, processing speed, and set-shifting in the first stage, and inhibitory and cognitive control in the latter task level (Langenecker et al., 2007). Cognitive flexibility and set-shifting were assessed using a 64 card Wisconsin Card Sorting task (Fox et al., 2013).

#### Cardiorespiratory Fitness

Participants completed a graded exercise test on a cycle ergometer (High-Performance Ergometer, Schoberer Rad MeBtechnik, Germany). Participants completed a 3-min warm-up at a self-selected cadence. The starting protocol commenced at 50 W for females (20W, *n* = 1) and 100 W for males (150W, *n* = 1) and was increased every 2 min by 10–30 W, to ensure participants reached maximal exertion ∼12–15 min after completion of the warm-up. Expired gases were collected using Hans-Rudolph facemasks and were analyzed as an average over 20 s to calculate the peak oxygen uptake (VO_2peak_) (Vyntus CPX Metabolic Cart, Jaeger, Germany). Heart rate (Polar Electro, Kempele, Finland) was monitored throughout each trial. The test ceased when participants were unable to maintain a cadence of >60 RPM for more than 30 s.

### Data Processing

Prior to data analysis, the SVIPT data were cleaned. Single trials were excluded if there were more than five force pulses and the incorrect order of colors was used, or if pulses were not accurately recorded. The primary outcome measure of motor-skill was calculated from the speed and accuracy of the force peaks in each trial. Speed was calculated as the time from the appearance of the colored targets, to the completion of the last pulse force of the trial. Trial force error is a measure of the sum of differences between a target, and the respective force peak of the participants attempt to hit the target over the five trials within a block. Subsequently, the average skill was calculated using the skill parameter calculation (Eq. 1), where a larger value represents better performance (Stavrinos and Coxon, 2017). Although the beta value in the below equation was developed in younger adults, it is similar to those previously utilized in older adults (Mooney et al., 2019), and was used across all participants, therefore not affecting differences in skill. The skill parameter has previously been log-transformed to reduce the heteroscedasticity of the data (Reis et al., 2009; Stavrinos and Coxon, 2017), however, sensitivity analysis revealed that this technique did not alter the results of the current study.

(1) $$Skillparameter=\frac{1-forceerror}{forceerror\cdot \left(\log{\left(duration\right)}^{1.627}\right)}$$

#### Statistical Analysis

Statistical analysis was conducted using R version 3.4.2 with statistical significance set at *p* < 0.05. The mean and standard deviation of measures taken at baseline were calculated for each group. One-way ANOVAs were utilized to compare the demographics, fitness, anxiety and depression symptomology, and cognitive battery scores of each group of participants at baseline. One-way ANOVA showed no group difference for skill at block one (*F*_2,38_ = 0.03; *p* = 0.97). A general linear mixed model with a random intercept fitted for subjects was utilized using the lme4 package to take into account the repeated measures design of the study. The model initially included the interaction effect between groups (exercise and a-tDCS, exercise and sham tDCS, and non-exercise and sham tDCS) and time [blocks 1–4, blocks 5–8, blocks 9–12, and retention (blocks 13 and 14)]. Non-significant interaction terms were dropped from the final model for ease of interpretation. Visual inspection of the QQ-plots generated for the models showed no significant deviation from normality. A type II Wald *F* test with the car package was utilized to obtain *p*-values for each model. Effect sizes are presented as the beta estimate from the general linear models for the relevant measures.

## Results

Due to a technical impedance error during the application of the a-tDCS, one participant from the exercise and active a-tDCS group was removed from the analysis. Further, one participant from the exercise and active a-tDCS group, and one from the non-exercise and sham tDCS were removed from the analysis after visual inspection of the data indicated a recording error with the motor-learning task. Forty-two participants (age = 44.6 ± 6.3, female = 32, and left handed = 3) had data available for analysis. There were no group differences at baseline in demographic characteristics, mood, fitness levels, or cognitive performance (Table 1). On average, participants had 17 years of education, and were university graduates. There was an average of 6 days between the final learning and retention session. At the completion of the retention session, participants were informed that some participants received sham-tDCS and were asked which form of tDCS they believed they received. Fifty-two percent of participants correctly reported which tDCS protocol was administered, which is similar to the chance-level of blinding reported in tDCS studies involving protocols of greater than 1 mA (Turi et al., 2019). Across the intervention sessions, the mean percent of age-predicted maximum heart rate (Tanaka et al., 2001) achieved by the end of the first epoch and the end of the fourth epoch were 85 ± 8 bpm and 90 ± 7 for the exercise and active a-tDCS group and 84 ± 5 and 90 ± 6 for the exercise and sham tDCS group.

**TABLE 1:** Group demographic, motor dexterity, self-reported mood, fitness, and cognitive performance characteristics and differences determined by one-way ANOVAs.

	**Exercise and sham tDCS *n* = 15**	**Exercise and active a-tDCS *n* = 13**	**Non-exercise and sham tDCS *n* = 14**	
	**Mean ± SD**	**Mean ± SD**	**Mean ± SD**	***p***
Age (years)	45 ± 5	43 ± 8	45 ± 6	*F*_2,39_ = 0.4;
				*p* = 0.70
Height (cm)	172 ± 10	175 ± 10	172 ± 9	*F*_2,39_ = 0.3;
				*p* = 0.72
Weight (kg)	73 ± 14	77 ± 11	72 ± 14	*F*_2,39_ = 0.5;
				*p* = 0.52
Female *n* (%)	11 (73)	11 (77)	10 (71)	-
Highest qualification*	6 ± 2	5 ± 2	6 ± 1	*F*_2,39_ = 0.3;
				*p* = 0.77
Education (years)	17 ± 3	17 ± 3	17 ± 4	*F*_2,39_ = 0.004;
				*p* = 0.99
Purdue pegboard sum of scores (no. of pegs)	56 ± 5	54 ± 6	57 ± 7	*F*_2,39_ = 0.8;
				*p* = 0.46
Goldberg anxiety score	0.5 ± 1.1	0.5 ± 0.7	0.6 ± 1.7	*F*_2,39_ = 0.03;
				*p* = 0.97
Goldenberg depression score	0.6 ± 1.8	0.6 ± 0.6	0.1 ± 0.3	*F*_2,39_ = 0.9;
				*p* = 0.42
MVPA (min week^–1^)	660 ± 1002	483 ± 506	407 ± 471	*F*_2,33_ = 0.4;
				*p* = 0.67
$\dot{\text{V}}$O_2_peak (mL min^–1^ kg^–1^)	40 ± 8	37 ± 11	40 ± 7	*F*_2,39_ = 0.7;
				*p* = 0.51
Stroop accuracy	100 ± 1	99 ± 3	100 ± 2	*F*_2,38_ = 2.6;
				*p* = 0.09
Stroop reaction time (ms)	752 ± 65	809 ± 132	741 ± 109	*F*_2,38_ = 1.6;
				*p* = 0.22
Wisconsin card sorting task correct (%)	73 ± 15	78 ± 10	75 ± 18	*F*_2,39_ = 0.5;
				*p* = 0.63
Wisconsin card sorting task reaction time (ms)	1901 ± 540	1747 ± 276	1814 ± 732	*F*_2,39_ = 0.3;
				*p* = 0.76
Go/No-Go task trial 1 accuracy (%)	96 ± 2	96 ± 3	94 ± 3	*F*_2,39_ = 1.6;
				*p* = 0.21
Go/No-Go task reaction time (ms)	476 ± 46	477 ± 32	480 ± 43	*F*_2,39_ = 0.05; *p* = 0.96
Go/No-Go task trial 2 accuracy (%)	94 ± 6	91 ± 9	88 ± 6	*F*_2,39_ = 2.1;
				*p* = 0.13
Go/No-Go task reaction time (ms)	493 ± 118	530 ± 79	536 ± 81	*F*_2,39_ = 0.9;
				*p* = 0.42

Presented as means and standard deviation. *Highest qualification, classified into qualification classes: 1 = high school, 2 = college, 3 = trade/vocational technical, 4 = diploma, 5 = bachelors, 6 = honors/graduate diploma, 7 = masters, 8 = doctorate.

### Online Change

**FIGURE 4:**
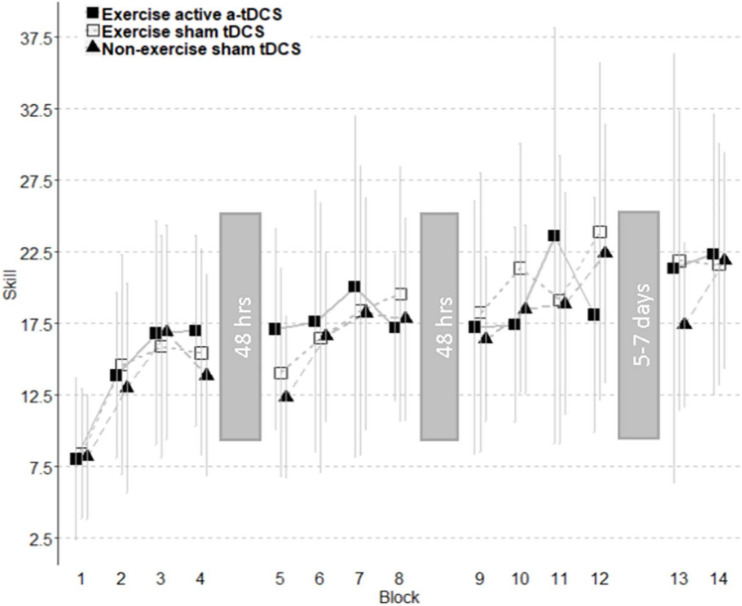
Improvement of motor-skill across all blocks. Skill change curves are separated into individual sessions with the time between sessions displayed within grayed blocks. The analysis of subcomponents showed an increase in motor skill over three intervention sessions observed across all groups. A trend toward a decrement of skill was observed between the first and second intervention session. Retention did not differ between groups. Error bars represent standard deviations.

**FIGURE 5:**
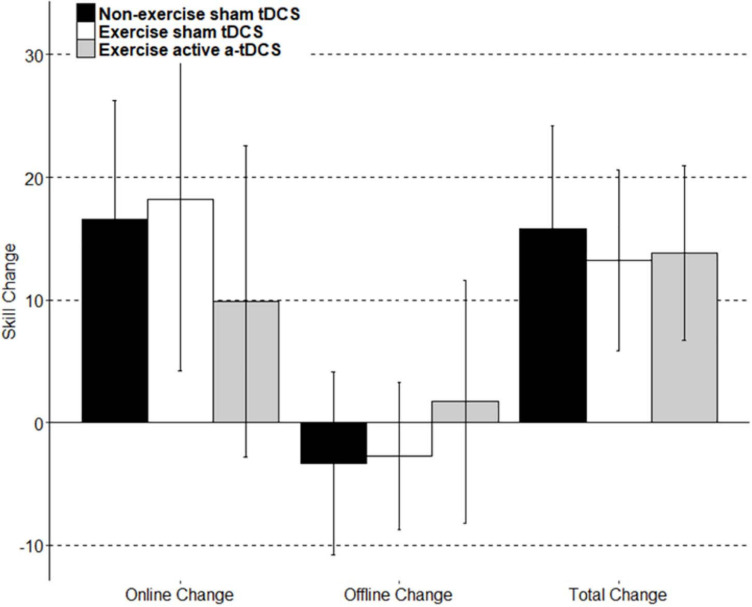
A visual representation of the group mean skill change score of subcomponents. Online change: the sum of difference of scores between only the last block and first block of each intervention session. Offline change: the sum of scores from the differences between only the first block of day two and the last block of day 1, and the first block of day three and the last block of day two. Total change: the sum of online change, offline change, the difference between the first block of retention and the last block of day 3, and the difference between the final retention block and the first retention block.

Online change refers to the change in motor-skill performance within a session, across blocks. This was assessed by comparing the average skill level of each block throughout the intervention sessions. As displayed in Figure 4, all three groups significantly improved their motor-skill level during the intervention period, demonstrated by a main effect of time (*F*_11, 444.08_ = 20.49; *p* = < 0.001), with skill increasing 15.55 (11.75–19.34 95% CI) between block 1 and block 12. However, there were no significant differences in motor-skill change between the groups across the intervention sessions, with no main effect of group (*F*_2, 39.01_ = 0.02; *p* = 0.98), and no group by time interaction (*F*_22, 422.08_ = 1.18; *p* = 0.26). Online change has been visually represented in Figure 5 as the sum of differences between the first and last training blocks of each intervention session [(Day1Block4 – Day1Block1) + (Day2Block4 – Day2Block1) + (Day3Block4 – Day3Block1)].

### Offline Change

Offline change refers to the difference in performance between training sessions. In this study, the offline change was assessed by comparing the average motor-skill level of the final block of each intervention session, with the first block of the following intervention session in separate models (Robertson et al., 2004; Reis et al., 2009). There was a trend toward a decrement in offline change observed between the final block of day one, and the first block of day two, with skill decreasing by −1.98 (−4.48 to 0.05 95% CI), however, the main effect of time was not significant (*F*_1,40.22_ = 38; *p* = 0.09), and did not differ between groups (*F*_2,39.01_ = 1.35; *p* = 0.27). There was no significant group by time interaction observed when assessing offline change between the final block of day one, and the first block of day two (*F*_2,38.25_ = 0.38; *p* = 0.69). Similarly, there was no main time effect observed for the offline change between the final block of day two and the first block of day three (*F*_1,41_ = 0.62; *p* = 0.44), and results did not differ significantly by group (*F*_2,39_ = 0.19; *p* = 0.83). There was also no group by time interaction effect (*F*_2,39_ = 0.38; *p* = 0.68). The results did not differ when an average of the final two blocks of an intervention session with the average of the first two blocks of the following intervention session. Offline change has been visually represented in Figure 5 as the sum of differences between the first training block of the second intervention session and the last training block of the first intervention session, and the first training block of the third intervention session and the last training block of the second intervention sessions [(Day2Block1 – Day1Block4) + (Day3Block1 – Day2Block4)].

### Retention of Motor-Skill

Retention of motor-skill was assessed by comparing the average skill of the four blocks from the final intervention session to the average skill of both blocks completed during the retention session. There was no main effect of time (*F*_1,39.23_ = 2.83; *p* = 0.10) or group (*F*_2,38.99_ = 0.07; *p* = 0.93). There was no significant group by time interaction observed (*F*_2,37.36_ = 0.23; *p* = 0.79). Total learning has been visually represented in Figure 5 to include the retention portion of the task. Total learning is represented as the sum of online change, offline change, and the difference between the first block of retention and the last block of learning (RetentionBlock1 – Day3Block4), and the difference between the first and last block of the retention session (RetentionBlock2 – RetentionBlock1).

Sensitivity analysis revealed that adding covariates of age, sex, fitness (relative VO_2_ peak), manual dexterity, cognitive function, the number of days between the intervention and retention session, and anxiety did not change the results of the study (all *p*’s > 0.05).

## Discussion

This study investigated the effects of a short-term high-intensity interval exercise intervention with and without a-tDCS applied over M1 on motor-skill change across three sessions, and retention 5–7 days later, in middle-aged adults. Although motor-skill learning occurred, there was no evidence of increased learning or retention when exercise was performed before the motor task, or when exercise was combined with a-tDCS. The current study is the first to our knowledge which has utilized high-intensity interval exercise and a-tDCS techniques within the one session targeting motor-skill learning, and one of the few investigations of motor learning in middle-aged adults.

The non-significant effects of high-intensity interval exercise and a-tDCS on motor learning in the current study should be discussed in the context of the study population. Older adults (60 years+) may have an impaired offline change when compared to young adults (Ehsani et al., 2015). However, middle-aged adults performed more similarly to younger than older adults on a perceptual motor-skill acquisition task (Kennedy and Raz, 2005). As the current middle-aged cohort was a highly educated, healthy population, it is unlikely that cognitive decline had occurred to a stage of offline change impairment. Previous literature has focused primarily on young adults (Reis et al., 2009; Mang et al., 2014; Stavrinos and Coxon, 2017), and although the current study shared several similarities with the intensity and timing of the high-intensity interval exercise (Mang et al., 2014; Stavrinos and Coxon, 2017) and a-tDCS protocols (Reis et al., 2009; Waters-Metenier et al., 2014) in relation to the motor task, interventions may need to be tailored to specific age and sociodemographic populations in future research.

High-intensity interval exercise did not improve the retention of a motor-skill 5–7 days after the intervention. This contrasts previous literature which has shown enhanced retention of motor-skill following high-intensity interval exercise (Roig et al., 2012; Skriver et al., 2014). The lack of benefit observed for a-tDCS on the retention of a motor-skill is in agreement with others who have similarly found no change in the long-term retention of a motor skill following a similar brain stimulation protocol, tested between eight and 30 days post learning (Reis et al., 2009). Previously, studies which have observed increased retention with high-intensity interval exercise (Roig et al., 2012; Skriver et al., 2014) have implemented multiple retention sessions. The repetitive testing at 1- and 24-h post-exercise may have provided additional consolidation opportunities, muddying the effects of exercise on retention. The consolidation of skilled finger movements is highly susceptible to interference, particularly within the first 6 h after learning a task (Krakauer and Shadmehr, 2006). This may have been exacerbated in the exercise group, as cognitive functions benefit from acute exercise for up to 2-h post-exercise (Basso et al., 2015). Thus, the exercise group may have received additional positive benefits from the exercise, when a 1-h consolidation was used. Further, it has previously been postulated that a 24-h retention session may have impacted the results seen at 48-h through additional consolidation (Korman et al., 2003). Future research should implement designs in which consolidation and retention are measured as separate entities to confirm the effects of exercise on the consolidation and retention of motor-skill learning. In the current study, no enhancement in a single retention session conducted 5–7 days after the motor-skill intervention was observed.

Online change was assessed by comparing the average skill level of each block throughout the learning phase. A positive online change was observed, however, there were no significant differences between groups. This is in line with previous literature, which has suggested that both a-tDCS during (Reis et al., 2009), and high-intensity interval exercise prior (Mang et al., 2014; Stavrinos and Coxon, 2017), to a task individually enhance learning through enhanced offline change, and not online change, of motor-skill learning in young adults.

Contrary to the hypothesis, the current study found no benefit of high-intensity interval exercise, or high-intensity interval exercise and a-tDCS to the offline phase of a motor-skill. Previously, exercise has improved the offline phase of a motor-skill in young adults after a single session of exercise (Mang et al., 2014; Stavrinos and Coxon, 2017). Previous studies which employed similar exercise protocols, tested the offline change of a motor-skill 5-h (Stavrinos and Coxon, 2017) and 24-h (Mang et al., 2014) after a single exposure to a motor-learning task, or a repeat exposure at a 24-h retention was utilized (Roig et al., 2012), whereas the current study assessed offline change between intervention sessions, which were 48-h apart. Enhancement in offline change by high-intensity exercise may be transient and therefore, may not have been observed in the current study due to the protracted time between sessions. Offline change may be influenced by the inclusion, or exclusion, of refamiliarization to the task, which may overcome the “warm-up decrement” sometimes observed in motor-learning literature (Adams, 1961). As there were no group differences between groups in the current study, it is unlikely that the exclusion of re-familiarization task impacted the outcomes. It should also be considered that in the current investigation comparisons were made to the first learning block. Future research may benefit from the inclusion of a baseline block prior to intervention. However, this can be difficult to balance as baseline testing may influence the observed learning curve.

The timing of the application of a-tDCS may have also contributed to the results of the current study. The current study applied a-tDCS during the motor task, which has previously enhanced the offline phase of motor-skill learning (Reis et al., 2009; Waters-Metenier et al., 2014). Exercise performed prior to a-tDCS has been hypothesized to provide an environment conducive to increased neuroplasticity (Mellow et al., 2020) due to an increase in the neuromodulator BDNF following exercise (Saucedo Marquez et al., 2015; Steinberg et al., 2019). Recent research, however, (Baltar et al., 2018) has demonstrated that a-tDCS applied prior to high-intensity exercise resulted in an inhibitory effect of cortical excitability for a 20-min period, suggesting that homeostatic plasticity may moderate the interaction between the interventions. Baltar et al. (2018) suggested that the high-intensity running protocol utilized may have caused fatigue, and therefore decreased cortical excitability, compared to the moderate-intensity running protocol, which caused excitatory effects. Recent evidence has also observed increases in motor-skill acquisition after moderate-intensity exercise (Statton et al., 2015). Cycling has been associated with improved motor and cognitive performance (Lambourne and Tomporowski, 2010) completed both during after exercise, including those designed to induce fatigue, and cognitive performance after exercise (Lambourne and Tomporowski, 2010). However, it may be that when combined with tDCS, in the current age population the high-intensity cycling may have induced a non-beneficial level of fatigue was induced. Perhaps in future works a lower exercise-intensity may be more beneficial. As this study did not measure motor evoked potentials, it cannot be confirmed whether the application of high-intensity exercise prior to a-tDCS had unexpected inhibitory effects. As there were no significant group effects, this was unlikely the leading cause for the non-significant results in the current study. Moderate-intensity exercise may be appropriate, as it has shown potential as a mechanism to enhance motor performance in older adults, proposed to be achieved through higher beta activity following the exercise (Hübner et al., 2018). Although the current investigation utilized a tDCS montage which has successfully enhanced motor sequence learning in a previous investigation (Waters-Metenier et al., 2014), modeling was not utilized to confirm the accuracy and specificity of the montage. tDCS montage modeling in future investigations is recommended to increase the specificity of tDCS application. One additional possibility is that following exercise, sweat on the scalp may have influenced the flow of current produced by tDCS (Horvath et al., 2014), by shunting the current away from the target area of the scalp and reducing the amount of stimulation to M1. While this cannot be ruled out, given benefit of tDCS applied during or after exercise on other cognitive functions (Ward et al., 2017), this is less likely to be the cause of the current findings. Although, the differences in application of tDCS between the current investigation should be considered. The use of a neoprene headcap and saline soaked electrodes, may have made the current set up more responsive to sweating compared to the discussed investigation (Ward et al., 2017) which utilized smaller, gel electrodes fixed by elastic band.

Although the non-significant effects of a-tDCS and high-intensity interval exercise on motor learning were unexpected in the current study, it is notable that two recent studies investigating the combination of a-tDCS and aerobic exercise on executive functions also reported no significant benefit on cognitive performance (Hendy et al., 2019; Thomas et al., 2020). While these studies had differences in protocol, including difference in cognitive tasks and stimulated regions, making them not directly comparable, these non-significant findings also reflect the ongoing difficulty in replicability of results using tDCS (Horvath et al., 2014), and to a more limited extent, aerobic exercise (Mellow et al., 2020). Individual differences in the response to tDCS have been observed (Filmer et al., 2019), and it may be that exercise induces individualized responses too (Hendy et al., 2019). These differences have been shown to include response modulation from base levels of gamma-aminobutyric acid and glutamate, known to relate to neurochemical excitability (Filmer et al., 2019), and the direction of response achieved (excitatory or inhibitory) (Wiethoff et al., 2014). However, as this investigation did not include a t-DCS only group, the effects of exercise on individual responses to tDCS cannot be determined. Further work is needed to establish the determinants of individual responses to both tDCS and aerobic exercise, both alone and in combination, before effective protocols can be potentially developed.

Although this study contained well-balanced groups, the samples high level of education, health, and larger proportion of female participants may limit the applicability of the findings to wider populations, as women may experience altered cortical excitability following stimulation dependent on the stage of their menstrual cycle (Inghilleri et al., 2004), and potentially lower excitation compared to men (Russell et al., 2017). However, the non-significant effect of sex within the models suggests this did not affect the outcome of the current study. Future research should extend to more comprehensive representations of the middle-aged adult population. Future research should also investigate the optimization of the timing between intervention sessions, and the timing at which exercise and a-tDCS are implemented within a session, as timing may be important in overcoming homeostatic plasticity. The addition of neurophysiology measures to investigate changes to cortical excitability and inhibition following these interventions, will further enhance future research in determining the interaction between exercise and non-invasive brain stimulation protocols. Additionally, investigations into the type of exercise, and the dose of a-tDCS will provide more insight into whether there is a beneficial combination for enhanced motor-skill learning in this population.

## Conclusion

The development and maintenance of motor-skills are a vital component of healthy aging. This study provides the first investigation of the effects of combining a short-term high-intensity interval exercise intervention with a-tDCS on a novel motor task in middle-aged adults. The current study adds to the limited literature regarding motor-skill learning in a middle-aged population, providing a methodological framework for future studies. Although the current study was not able to confirm the prescription of exercise required to enhance motor-skill learning in middle-aged adults, it demonstrated that short-term intervention consisting of three 20-min high-intensity interval exercise sessions did not increase motor-skill learning or retention 5–7 days later, either individually, or when paired with a-tDCS applied during a motor-learning task compared to a control group.

## Data Availability Statement

The raw data supporting the conclusions of this article will be made available by the corresponding author, without undue reservations.

## Ethics Statement

The studies involving human participants were reviewed and approved by the University of Canberra and the Australian National University Human Research Ethics Committee (HREC 16-121, and 2016/322, respectively). The patients/participants provided their written informed consent to participate in this study.

## Author Contributions

CQ: conceptualisation, methodology, investigation, formal analysis, data curation, and writing – orgininal draft preparation. BR, DP, and JN: conceptualisation, methodology, formal analysis, and writing – orgininal draft preparation. JC: conceptualisation, methodology, formal analysis, data curation, writing – review and editing, and software. NC: conceptualisation, methodology, and writing – review and editing. SA: conceptualisation, methodology, investigation, formal analysis, and writing – review and editing. All authors contributed to the article and approved the submitted version.

## Conflict of Interest

The authors declare that the research was conducted in the absence of any commercial or financial relationships that could be construed as a potential conflict of interest.
